# Biofilm growth and IL-8 & TNF-α-inducing properties of *Candida albicans* in the presence of oral gram-positive and gram-negative bacteria

**DOI:** 10.1186/s12866-020-01834-3

**Published:** 2020-06-11

**Authors:** Radhika G. Bhardwaj, Arjuna Ellepolla, Hana Drobiova, Maribasappa Karched

**Affiliations:** Oral Microbiology Research Laboratory, Department of Bioclinical Sciences, Faculty of Dentistry, Kuwait University, PO Box 24923, 13110 Safat, Kuwait

**Keywords:** *Candida albicans*, Polymicrobial biofilms, qPCR, Gram-positive bacteria, Gram-negative bacteria, Cytokines

## Abstract

**Background:**

Interaction of *C. albicans* with oral bacteria is crucial for its persistence, but also plays a potential role in the infection process. In the oral cavity, it grows as part of dental plaque biofilms. Even though growth and interaction of *C. albicans* with certain bacterial species has been studied, little is known about its biofilm growth in vitro in the simultaneous presence of Gram-negative and Gram-positive bacteria. The aim was to evaluate the growth of *C. albicans* in polymicrobial biofilms comprising oral Gram-negative and Gram-positive bacteria. Further, we also aimed to assess the potential of *C. albicans* in the *Candida*-bacteria polymicrobial biofilm to elicit cytokine gene expression and cytokine production from human blood cells.

**Results:**

*C. albicans* cell counts increased significantly up to 48 h in polymicrobial biofilms (*p* < 0.05), while the bacterial counts in the same biofilms increased only marginally as revealed by qPCR absolute quantification. However, the presence of bacteria in the biofilm did not seem to affect the growth of *C. albicans*. Expression of IL-8 gene was significantly (*p* < 0.05) higher upon stimulation from biofilm-supernatants than from biofilms in polymicrobial setting. On the contrary, TNF-α expression was significantly higher in biofilms than in supernatants but was very low (1–4 folds) in the monospecies biofilm of *C. albicans*. ELISA cytokine quantification data was in agreement with mRNA expression results.

**Conclusion:**

Persistence and enhanced growth of *C. albicans* in polymicrobial biofilms may imply that previously reported antagonistic effect of *A. actinomycetemcomitans* was negated. Increased cytokine gene expression and cytokine production induced by *Candida*-bacteria polymicrobial biofilms and biofilm supernatants suggest that together they possibly exert an enhanced stimulatory effect on IL-8 and TNF-α production from the host.

## Background

*Candida albicans* is a commensal fungus that colonizes the oral cavity and various other sites in human body. Its extensive interaction with oral bacteria might be crucial for its persistence but also potentially contributes to infection process [1]. Despite that complex microbial interactions in dental plaque biofilm have been extensively studied during the past 10–15 years, inter-kingdom interactions have received little attention [2].

*C. albicans* coexists with a multitude of bacterial species [3] and its interactions with streptococci are often mutually beneficial for their survival in diverse oral niches [2]. On the other hand, not much is known of the interaction between *C. albicans* and the Gram-negatives such as *Aggregatibacter actinomycetemcomitans*. Intriguingly, autoinducer 2 (AI-2) of *A. actinomycetemcomitans* inhibits *C. albicans* biofilm formation [4], while the same factor produced by streptococci has the opposite effect [5], suggesting differences in roles of the same quorum sensing factors released by each bacterial species.

A critical aspect of microbial infections is the provocation of the host cells to produce inflammatory cytokines. The ability of various *Candida* species and their biofilms to induce cytokine production from host cells has been reported in a number of studies [6–8] and a prominent role of key cytokines such as TNF-α and IL-8 in candidiasis has been known [7, 9]. Concurrently, diverse oral bacteria and their biofilms orchestrate host cytokine response as documented by a large number of in vitro and in vivo studies [10–14]. In the oral cavity, while encased in complex plaque biofilms, microorganisms can release their cellular components, which can breach host barriers impermeable to whole microbial cells, reach distant sites in the host body and cause tissue destruction. We and others have previously investigated the potential of bacterial biofilm supernatants containing secreted soluble components, in addition to biofilms, to cause cytokine production from host cells [14–17]. Proteomic analyses of biofilm-supernatants has revealed the presence of virulence-related proteins among other secreted proteins [18, 19]. However, there is a dearth of knowledge on cytokine-inducing potential of *Candida*-bacteria polymicrobial biofilms and biofilm-supernatants containing soluble secreted components from biofilms. While recent research has thrown more light on interactions operating between bacteria and *Candida* [20–22], little is known how *Candida* grows in the simultaneous presence of Gram-positive and Gram-negative bacterial partners in polymicrobial biofilms. Further, we also aimed to investigate cytokine gene expression and cytokine production from human blood cells upon challenge with *Candida*-bacteria polymicrobial biofilms.

## Results

### Biofilm formation by *Candida* and the bacterial species

**Fig. 1:**
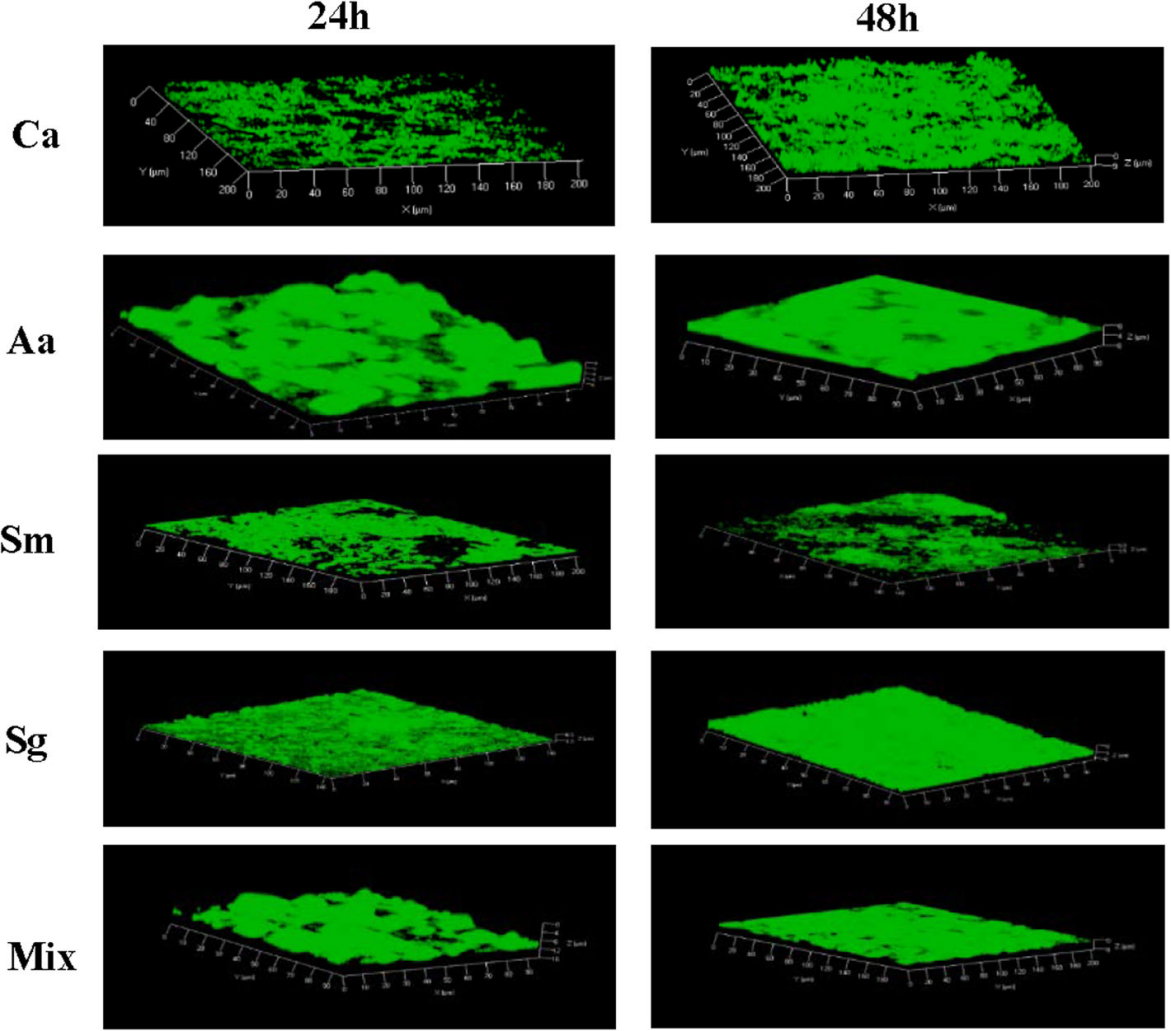
Confocal laser scanning microscopy 3-dimensional (3D) images of monospecies and polymicrobial biofilms of bacteria and *C. albicans*. *A. actinomycetemcomitans* (Aa), *S. mutans* (Sm), *S. gordonii* (Sg) and *C. albicans* (Ca) were cultured as monospecies and polymicrobial (mix) in brucella broth in the wells of Millicell® EZ slides (Millipore) in aerobiosis in 5% CO_2_ at 37 °C for 24 and 48 h and stained with Syto9®. Images were acquired on Carl-Zeiss LSM 700 at × 630 magnification and 3D view reconstructed using the software ZEN 2012

*C. albicans* and all the test bacteria formed biofilms as evident from confocal laser scanning microscopy images (Fig. 1). The 3-dimensional images in Fig. 1 showed the formation of mat-like biofilms by *C. albicans* and the three test bacteria individually as well as when grown together.

### qPCR quantification of *Candida* and bacteria in monospecies and polymicrobial biofilms

**Fig. 2:**
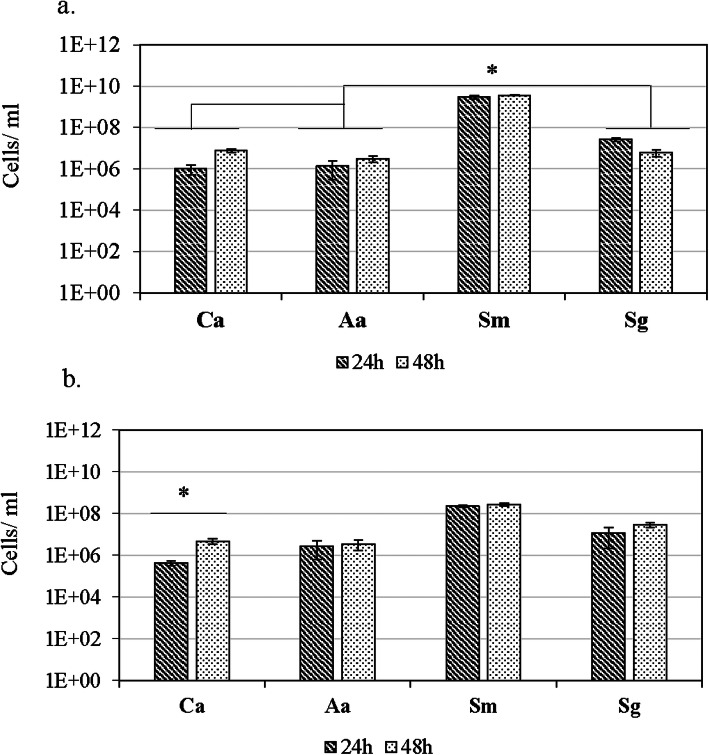
Quantities of bacterial species and *C. albicans* grown as monospecies and polymicrobial biofilms. Standardized numbers of the four species were added to 24-well cell culture plates for monospecies (**a**) and polymicrobial biofilm (**b**). The cultures were incubated for 24 and 48 h. At the end of each time point, bacterial viability was checked by plating a small aliquot on brucella blood agar. Bacterial quantities (cells/ml) were determined by using qPCR SYBR Green assay. The results shown are median values from three independent experiments. Abbreviations: Ca = *C. albicans*, Aa = *A. actinomycetemcomitans*, Sm = *S. mutans*, Sg = *S. gordonii*

qPCR showed that *C. albicans* and all the test bacterial strains grew as monospecies biofilms till 48 h of incubation period. In monospecies biofilm, *C. albicans* median cells per ml increased 7-fold from 9.8 × 10^5^ at 24 h to 6.4 × 10^6^ after 48 h. Among the bacterial species, *S. mutans* quantities were highest (*P* < 0.05): 3 × 10^9^ cells per ml at 24 h and remained nearly the same (3.7 × 10^9^) after 48 h. *S. gordonii* biofilm showed median cells per ml 2.6 × 10^7^ at 24 h and slightly decreased to 6 × 10^6^ after 48 h. Median counts of *A. actinomycetemcomitans* were 10^6^ cells per ml at 24 h and slightly increased to 3 × 10^6^ cells per ml after 48 h (Fig. 2a).

In polymicrobial biofilms, qPCR revealed the presence of all the four test species till 48 h of incubation (Fig. 2b). There was a significant (*P* < 0.05) increase in *C. albicans* median cells per ml: 13-fold from 3.7 × 10^5^ at 24 h to 4.7 × 10^6^ at 48 h. While *S. mutans* cell numbers did not seem to increase after 24 h, median cells per ml were about 1 log lower compared to the cell numbers in monospecies biofilm. In polymicrobial biofilms, from 24 h to 48 h, *S. gordonii* cells increased 3-fold whereas the increase in cell numbers was only one fold each in *S. mutans* and *A. actinomycetemcomitans*.

### Cytokine gene expression in biofilm- and biofilm-supernatant stimulated human blood cells

**Fig. 3:**
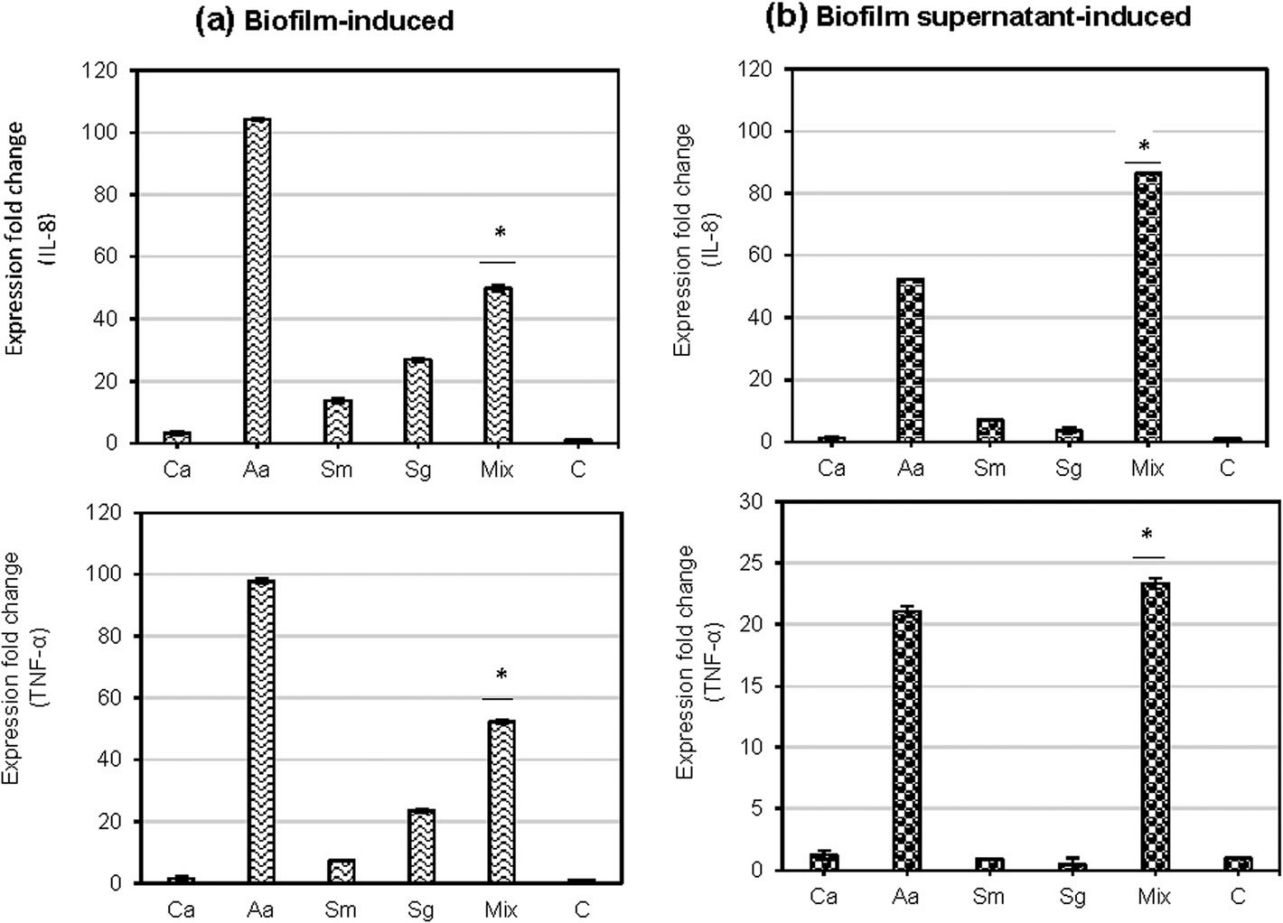
mRNA expression of cytokine genes by biofilms (**a**) and biofilm supernatants (**b**). Human blood cells were stimulated by monospecies and polymicrobial biofilm (**a**) and biofilm supernatants (**b**) of bacteria and *C. albicans*. Abbreviations: Ca = *C. albicans*, Aa = *A. actinomycetemcomitans*, Sm = *S. mutans*, Sg = *S. gordonii*, Mix = Mixture, C=Control, *p < 0.05

Upon stimulation of blood cells with supernatants from *Candida*-bacteria polymicrobial biofilm, significantly (*P* < 0.05) higher fold change in expression of IL-8 genes was observed (86-folds, respectively) than biofilms (50-folds). On the contrary, TNF- α was significantly (P < 0.05) higher in biofilms (52-fold) than in supernatants (23-fold) (Fig. 3).

Among bacterial biofilms, *A. actinomycetemcomitans* biofilm induced highest levels of IL-8 and TNF- α, as evident from ~ 100-fold (IL-8, TNF- α) increase in mRNA expression upon stimulation (Fig. 3a). *S. gordonii* biofilm, but not the supernatant, consistently induced higher folds of all cytokine mRNA expression than *S. mutans* (Fig. 3a). Cytokine gene expression fold change was very low (in the range of 1–4 folds) in blood cells stimulated with *C. albicans* monospecies biofilm.

### Quantification of select cytokines by ELISA

**Fig. 4:**
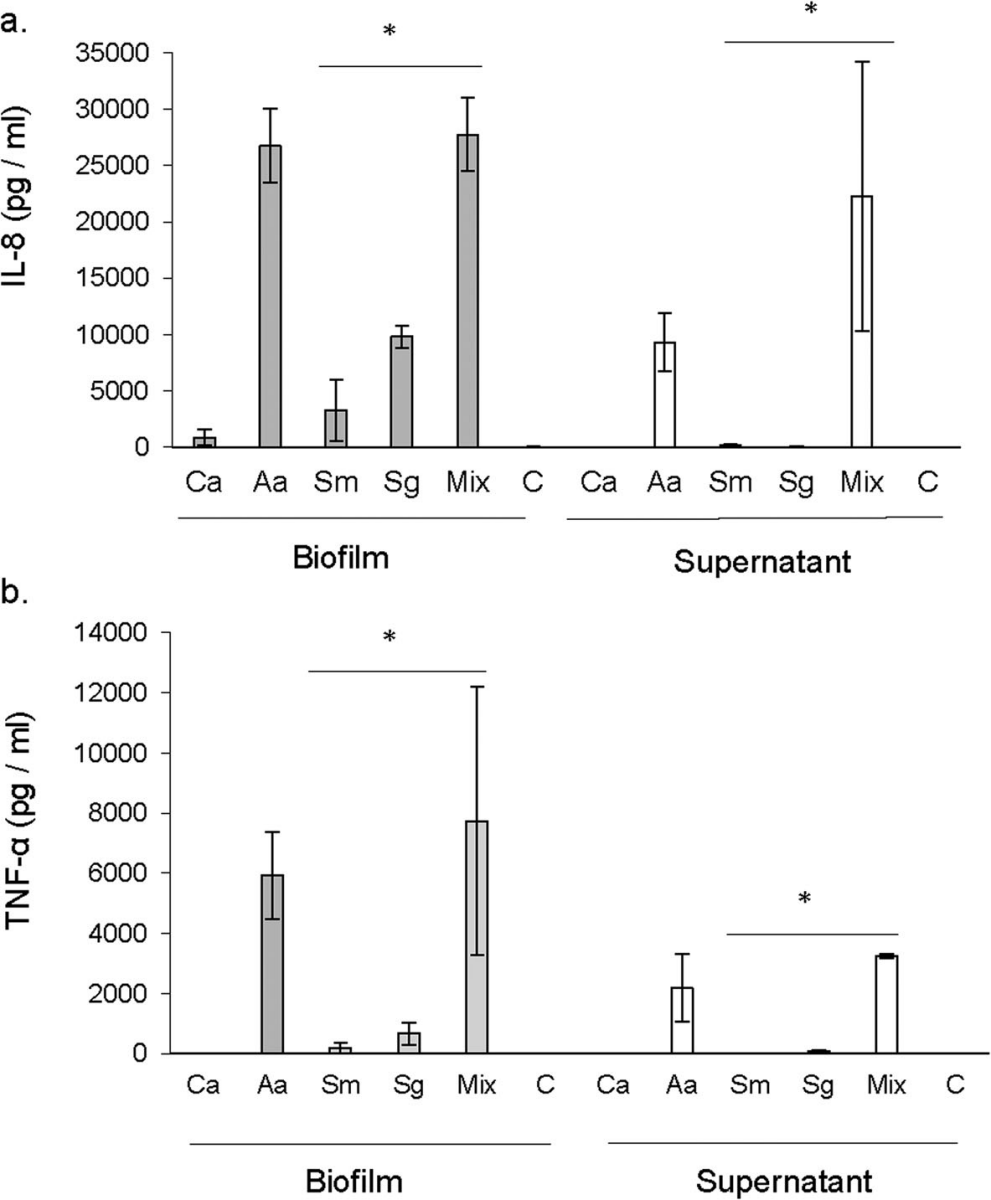
IL-8 and TNF-α induction by biofilms and biofilm supernatants. Human blood cells were stimulated by 24-h old monospecies and polymicrobial biofilms and biofilm supernatants. The stimulated samples were analyzed by ELISA for the quantification of IL-8 (**a**) and TNF-α (**b**). Ca = *Candida albicans*; Aa = *Aggregatibater actinomycetemcomitans*; Sm = *Streptococcus mutans*; Sg = *Streptococcus gordonii*; Mix = Mixture and C=Control, *p < 0.05

Mean (SD) levels of IL-8 induced by *C. albicans* biofilm were 865 (699) pg/ml while *C. albicans* biofilm-supernatant induced a very low amount of 20 pg/ml. *A. actinomycetemcomitans* biofilm induced highest levels of IL-8, 26,781 (3307) pg/ml, while the biofilm-supernatant from the same species induced 9354 (2600) pg/ml. *S. gordonii* and *S. mutans* did show induction, 9859 (987) and 3315 (2751) pg/ml, respectively but, the levels were significantly (*P* < 0.05) lower than those induced by the polymicrobial biofilm and the respective supernatant. Biofilm supernatants from *S. gordonii* and *S. mutans* failed to induce IL-8. Highest IL-8 levels were induced by biofilms [27,786 (3248) pg/ml] and supernatants [22,312 (11939) pg/ml] of *Candida*-bacteria polymicrobial biofilm in comparison to monospecies cultures (Fig. 4a).

Similarly, the levels of TNF- α (Fig. 4b) were significantly higher in polymicrobial biofilm by both, biofilms [7747 (4450) pg/ml] and biofilm-supernatants [3246 (65) pg/ml], in comparison to monospecies biofilms of *S. mutans* and *S. gordonii*. TNF- α was under detection levels in both the *C. albicans* biofilm and biofilm-supernatant stimulated blood cells. Among monospecies biofilms, *A. actinomycetemcomitans* showed the highest induction levels of TNF- α [5936 (1454) pg/ml for biofilms and 2186 (1129) pg/ml for supernatants]. Biofilm supernatants from *S. gordonii* and *S. mutans* failed to induce TNF- α.

## Discussion

This study showed that *C. albicans* did not only persist in the polymicrobial biofilm in the simultaneous presence of Gram-positive and Gram-negative bacteria, but also its growth was significantly enhanced. The biofilms and biofilm-supernatants from 24-h old *Candida*-bacteria polymicrobial biofilm cultures induced significantly higher levels of gene expression as well as production of the two important cytokines IL-8 and TNF- α. *C. albicans* monospecies biofilm or the biofilm-supernatant consistently induced only very low levels of these cytokines.

Our qPCR results from the species quantification of *Candida*-bacteria polymicrobial biofilm cultures showed that each of the *Candida* and the bacterial species tested were able to grow and persist in the polymicrobial biofilms. Importantly, quantities of *C. albicans* increased in the polymicrobial biofilm, suggesting that the interactions among these species within biofilms perhaps benefitted *Candida* growth. Although, different oral bacterial species have been investigated in dual/mixed biofilms with *C. albicans* in vitro, [2, 23–25] the combination of *A. actinomycetemcomitans* (Gram-negative) and streptococci (Gram-positives) with *C. albicans* has not been tested before. We chose the Gram-negative species *A. actinomycetemcomitans* because of its established implication in periodontitis [26–29]. *S. mutans* is a major etiologic agent in dental caries while *S. gordonii* is more associated with health (caries-free) [30–35]. With the known antagonism between *A. actinomycetemcomitans* and streptococci [36], we were interested in investigating how *C. albicans* would fare in a polymicrobial biofilm consisting the latter microorganisms. It has been demonstrated that *C. albicans* presence can enhance *S. mutans* growth, accumulation, and biofilm formation both in vitro and in vivo [37–43]. *S. mutans* displayed a strong affinity to *C. albicans* hyphae, as shown by electron microscopy on both human teeth and hydroxyapatite substrate samples [41]. Existing literature suggests that biofilm interactions of *C. albicans* with *A. actinomycetemcomitans* or streptococci are complex. For example, *A. actinomycetemcomitans* produce autoinducer-2 (AI-2), which inhibits *C. albicans* biofilm formation [4]. However, AI-2 produced by *S. gordonii*, increase biofilm formation and filamentation of *C. albicans* [5]. A small peptide called competence-stimulating peptide (CSP), produced by *S. mutans*, but not by *S. gordonii*, inhibits *C. albicans* hyphal formation in the early stages of biofilm formation [44, 45].

In distinct contrast to earlier studies where *C. albicans* biofilms involving streptococci or *A. actinomycetemcomitans* showed either antagonistic or synergistic growth behavior [4, 46], all microbial species tested in our in vitro model grew and persisted in biofilms until 48 h. At this phase we do not know how mutual antagonism was negated in the current *Candida*-bacteria polymicrobial biofilms. However, one could speculate that *C. albicans* might regulate antagonistic interactions between *A. actinomycetemcomitans* and streptococci while evading itself from the inhibitory effect of *A. actinomycetemcomitans* AI-2, possibly in an indirect way, by taking advantage of *S. gordonii* AI-2 which enhances *C. albicans* biofilm [5].

We investigated the inflammatory potential of biofilms and biofilm-secreted components from *C. albicans* and *Candida*-bacteria polymicrobial biofilms. This was done at two levels: mRNA and protein. We used human whole blood from a healthy subject because of its easy access and previous use in immunological studies in oral pathogens [6, 47]. The cytokines in this study were chosen because of their significance in oral infections such as periodontitis [48, 49] and caries [50, 51]. IL-8 and TNF- α play a crucial role in innate immune response. While IL-8 is a known neutrophil recruiter, TNF-α triggers robust host immune response by promoting infiltration of inflammatory cells and can directly contribute to osteoclast genesis and bone resorption [52]. In our study, IL-8 and TNF-α were elicited highest in *Candida*-bacteria polymicrobial biofilm and biofilm-supernatant stimulated blood in comparison to monospecies cultures. Only the bacterial monospecies biofilm cultures, not *C. albicans*, showed an induction of TNF- α**.** Contrary to our findings, *C. albicans* was reported as a better stimulator of TNF- α from blood cells of healthy subjects [6]. The reason that *C. albicans* stimulated very low levels of cytokines repetitive experiments in our study is possibly variability in responses from individuals. The variations in cytokine production by mononuclear cells in various healthy human volunteers, irrespective of age have been reported earlier [53]. Moreover, host (age, gender etc.) and environmental factors influencing human cytokine responses further supports cytokine response variability in humans [53, 54].

The expression of the genes encoding IL-8 and TNF-α was investigated by real time PCR. Our results are in line with several studies that demonstrated enhanced expression of host cytokine genes upon stimulation by oral microorganisms including *C. albicans*, *A. actinomycetemcomitans, S. mutans* and *S.gordonii* [6, 7, 13, 14, 55–59]. While IL-8 gene was more induced by the supernatant than the biofilm in the polymicrobial biofilms, it was the opposite in the case of TNF-α gene. Supernatants of *Candida*-bacteria polymicrobial biofilms were found to induce highest fold change in the expression of cytokine genes than did the supernatants of monospecies biofilms. In a polymicrobial biofilm setting, cell components of one species might affect the cytokine-stimulating potential of other species. For example, *S. sanguinis* peptidoglycan inhibited the cytokine expression induced by the LPS of periodontopathogens due to the inhibition of LPS binding to LBP and CD14 [55].

An interesting observation was that *A. actinomycetemcomitans*, which induced highest inflammatory response among monospecies biofilms, appeared to have a lesser immunostimulatory components in its biofilm-supernatants than the biofilms. Even though in silico analysis of the *A. actinomycetemcomitans* biofilm secretome revealed the presence of a large number of virulence related proteins [18], in our study, the biofilm-secreted components were yet less effective than the biofilm in inducing IL-8 and TNF-α.

In general, the cytokine gene expression results were in agreement with cytokine ELISA quantification. *Candida*-bacteria polymicrobial biofilms induced higher TNF-α, both at the gene level and at the protein level, than did the respective biofilm-supernatants. The polymicrobial biofilm did induce higher IL-8 than the supernatants at the protein level, but, the expression of IL-8 gene was more in the supernatants compared to biofilms. Further, supernatants from polymicrobial biofilms always triggered more cytokine gene expression and cytokine production than the supernatants from monospecies biofilms, suggesting an enhanced ability to induce host cell inflammatory response, of the microbial secretion products when existing together in the supernatants.

## Conclusions

*C. albicans* grew and persisted as part of polymicrobial biofilms containing the Gram-negative bacterium *A. actinomycetemcomitans* and the Gram-positive species *S. mutans* and *S. gordonii*. These data suggest that hitherto known antagonistic effect of *A. actinomycetecomitans* on *C. albicans* growth [4] was negated in the current polymicrobial biofilm setting possibly due to the presence of streptococci. Further, induction of higher levels of certain cytokines and an increased expression of corresponding cytokine genes suggests a possibility that such *Candida*-bacteria polymicrobial biofilm communities together derive a cumulative potential to exert increased cytokine stimulatory effect on the host.

## Methods

### Microbial strains and culture conditions

Reference bacterial strains *Streptococcus mutans* CCUG 11877 T, *Streptococcus gordonii* CCUG 33482 and a yeast strain *Candida albicans* ATCC 24433 were purchased from culture collections. *Aggregatibacter actinomycetemcomitans* strain SA269 was from the strain collection of Sirkka Asikainen (Umeå University, Sweden). Bacterial strains were cultured on brucella blood agar (BBA) for 2 days and *C. albicans* on sabouraud dextrose agar (SDA) for 24 h in 5% CO_2_ in air at 37 °C

### Biofilm cultures

Established method [60] with some modifications was used for culturing biofilms. First, an inoculum of OD_600_ = 1 suspension of each test species was prepared. For this the bacterial/yeast colonies were harvested from agar plates with sterile plastic loops and suspended in sterile PBS. The suspensions were washed two times in sterile PBS by centrifuging at 5000×g for 5 min. The cell pellet recovered after centrifugation was suspended in 1-ml brucella broth. Based on the OD measurements of the above suspensions, each test species was adjusted to a standard OD_600_ = 1. As determined in our laboratory, at OD_600_ = 1, the CFU/ml were for *A. actinomycetemcomitans* 1 × 10^8^, *S. mutans* 1.7 × 10^8^, *S. gordonii* 1.6 × 10^8^ and *C. albicans* 6.7 × 10^6^. Further these suspensions (OD_600_ = 1) were used to culture monospecies and polymicrobial biofilms in 24-well culture plates. Monospecies biofilms were initiated by inoculating 24-well plates containing 900 μl brucella broth with a 100 μl aliquot from the above OD_600_ = 1 suspension of each species. For polymicrobial biofilm culture, inoculum was prepared in a microfuge tube by combining equal volumes of OD_600_ = 1 suspensions from each of the four test species. One hundred microliter from this species mixture was transferred into the wells of 24-well plates containing 900 μl of brucella broth. Total three sets of monospecies and polymicrobial biofilms were cultured in parallel. Two sets were cultured in 24-well culture plates for analysis of mRNA expression levels of cytokine genes in human blood cells stimulated with biofilm and biofilm-supernatant, ELISA based quantification of selected cytokines and qPCR quantification of biofilms while third set was cultured on Millicell® EZ slides (EMD Millipore) with detachable wells for confocal laser scanning microscopy of biofilms. For qPCR quantification of biofilms, at the end of the incubation time points, supernatant broth was aspirated and the biofilms were washed once with sterile PBS to remove unattached cells. The biofilms were thoroughly scraped off and the wells were further examined under stereomicroscope to confirm the complete removal of biofilms. Harvested biofilms were resuspended in 100 μl sterile nuclease-free H_2_O (Ambion, USA) and then immediately preserved at − 20 °C until used for DNA extraction.

### Confocal laser scanning microscopy of biofilms

Monospecies and polymicrobial biofilms were cultured on Millicell® EZ slides (EMD Millipore) with detachable wells [15]. 24-and 48 h old biofilms were washed twice with sterile PBS (1 ml) to remove loosely attached and unbound bacteria and/or *Candida* cells and were then fixed for 30 min at room temperature with 4% freshly prepared paraformaldehyde in PBS (1 ml/cell, pH 7). The biofilms were washed in PBS and stained with Syto® 9 Green Fluorescent Nucleic Acid Stain (Molecular Probes) for 15 min at room temperature in dark according to the manufacturer’s recommendations. The biofilms were washed in PBS again and the polypropylene wells were then detached and the slides were air-dried. The biofilms were covered with a cover glass containing a drop of BacLight® mounting oil. Confocal laser scanning microscope (LSM 700, Carl-Zeiss; software ZEN 2012) was used to analyze and capture the images of stained biofilms. Three dimensional reconstruction of the biofilms was done using Z-stack.

### DNA extraction and purifications

DNA from the harvested biofilms was purified using DNeasy DNA extraction kit (Qiagen). Enzymatic lysis buffer containing Tris EDTA buffer (20 mM Tris, 2 mM EDTA) with 1.2% Triton X-100 and lysozyme was used. Purified DNA was eluted in nuclease free water and concentrations were measured by UV spectrophotometry method using NanoDrop™ 1000.

### Quantifying biofilms by qPCR

For qPCR quantification, Species-specific primers for the bacterial 16S rRNA gene and internal transcribed spacer regions 1 of rRNA and universal reverse primer of 26 s rRNA gene of *C. albicans* were used (Table 1). The reaction mixture containing 12.5 μl SYBR Green master mix (Power SYBR Green® Kit, Applied Biosystems), 1.0 μl each of forward and reverse primer (0.4 μM), 5.0 μl DNA template (equivalent to 50 ng DNA) and 5.5 μl H_2_O was used in qPCR. A thermal profile with an initial denaturation at 95 °C for 10-min, 40 cycles of 95 °C for 15 s – 30 s, 52–68 °C (depending on the primer pair) for 30 s and 72 °C for 30 s - 1 min was run for carrying out amplifications on ABI Fast RT-PCR machine. The elongation step was considered for the fluorescent signal acquisition. Software SDS v2.3 was used for data analysis. Serially diluted DNA from the above species in the reaction and plotting the Ct values against deduced bacterial cell concentration (cells/ml) for each species were used to construct standard curves using the above software. The standard curves were generated in the above RT-PCR software by computing the bacterial cell numbers. The optimum reaction efficiency of 86–105% (slope − 3.7 to − 3.2) and R^2^ value (0.97–0.99) for standard curve linearity were considered.

**Table 1:** Primers and conditions used in qPCR quantification of microbial cultures

Species	Primer sequence 5′-3′	Annealing temperatures (°C)	References
*A. actinomycetemcomitans*	F: TAGCCCTGGTGCCCGAAGC R: CATCGCTGGTTGGTTACCCTCTG	68	[61]
*S. mutans*	F: TCGCGAAAAAGATAAACAAACA R: GCCCCTTCACAGTTGGTTAG	56	[62]
*S. gordonii*	F: GGTGTTGTTTGACCCGTTCAG R: AGTCCATCCCACGAGCACAG	53	[63]
*C. albicans*	F: TCA ACT TGT CAC ACC AGA TTA TT R: TCC TCC GCT TAT TGA TAT GC	52	[64]

### Stimulation of human blood cells by monospecies biofilms and *Candida*-bacteria polymicrobial biofilms

Ethical approval for blood collection from a healthy human volunteer was obtained from Health Science Center Ethical Committee, Kuwait University. One ml of whole blood from a healthy human volunteer was added to the 24-h old biofilms in each well and incubated for 24 h at 37 °C in 5% CO_2_ in air for stimulation. Well with only blood and no biofilm was used as control. For stimulation with biofilm supernatants, broth supernatants from 24 h old biofilms were filtered through 0.2 μm syringe filters to get rid of the bacterial cells. Two hundred microliters of the filtered supernatant was added into wells containing 1 ml blood. After 24 h of incubation, samples were taken out and centrifuged immediately at 5000×g for 5 min. The supernatant was stored at − 20 °C until use.

### Analysis of mRNA expression levels of cytokine genes in human blood cells stimulated with biofilms and biofilm-supernatants

To determine the mRNA expression of IL-8 and TNF-α genes, total cellular RNA was extracted and purified from stimulated blood samples as per manufacturer’s instructions (QIAamp RNA blood Mini kit**)**. Erythrocytes were lysed in buffer EL by incubating on ice for 15 min, vortexing and centrifugation at 400×g at 4 °C for 10 min. Non-nucleated lysed erythrocytes in the supernatant were discarded while leukocytes in the pellet were further disrupted in RLT buffer. The lysate was homogenized by centrifugation at maximum speed in QIAshredder spin column and then precipitated with 70% ethanol and transferred to QIAamp spin column (provided in the kit). The RNA bound to the membrane was further treated with buffers RW1 and RPE and discarded the flow through. Finally bound RNA was eluted in RNase-free water. The concentration of RNA was determined by spectrophotometer NanoDrop™ 1000 and the purity of RNA was assessed by A260/A280 ratio. Purified RNA was converted to cDNA by using High-Capacity cDNA Reverse Transcription Kit (Applied Biosystems™) according to manufacturer’s instructions. The concentration of cDNA was determined by spectrophotometer NanoDrop™ 1000 and the purity of DNA was assessed by A260/A280 ratio.

### Reverse trascriptase RT-PCR

A reaction mixture of 10 μl SYBR Green master mix (Power SYBR Green® Kit, Applied Biosystems), 1.0 μl each of forward and reverse primer (0.4 μM), 7 μl H_2_O and 1.0 μl cDNA template (equivalent to 50 ng cDNA) was used to perform Real Time reverse trascriptase PCR reaction on ABI 7500 Fast Real-Time PCR machine. Thermal profile of 10-min initial denaturation at 95 °C, 40 cycles of 95 °C for 15 s – 30 s, 50–60 °C for 30 s (depending on the primer pair) and 72 °C for 30 s – 1 min were run to carry out the amplifications. Primers and conditions used in qPCR for mRNA gene expression analyses are mentioned in Table 2. Expression levels of all the target genes were normalized using endogenous housekeeping gene encoding GAPDH. Fluorescent signal acquisition was set at the elongation step. Data analysis was accomplished using the software SDS v2.3. Analysis of the expression of cytokine controlling genes was performed on cells from untreated control, biofilm and biofilm supernatant treated groups by comparative ∆∆Ct method in ABI 7500 SDS system (Applied Biosystems). The amount of target, normalized to an endogenous reference (GAPDH) and relative to a calibrator (untreated), was given by 2^_∆∆Ct^ and the expression fold change was presented graphically.

**Table 2:** Primers and conditions used in RT-PCR for cytokine gene expression analysis

Target gene	Primer sequence 5′-3′	Size (bp)	Annealing temperatures (°C)	References
TNF-α	F: GAAACCTGGGATTCAGGAATG R: GTCTCAAGGAAGTCTGGAAAC	246	55	[56]
IL-8	F: GCAGCTCTGTGTGAAGGTGCAG R: GCATCTGGCAACCCTACAACAG	365	55	[58]
GAPDH	F: ATCACCATCTTCCAGGAG R: ATCGACTGTGGTCATGAG	318	55	[59]

### ELISA based quantification of selected cytokines

For cytokine quantifications, ELISA immunoassay kits (Quantikine® ELISA R&D systems) containing a cytokine specific monoclonal antibody pre-coated ELISA plates were used. The assay was based on a quantitative sandwich enzyme immunoassay technique. To determine the absolute quantities of IL-8 and TNF-α in blood upon stimulation with biofilm or biofilm-supernatant, the wells of ELISA plate were pipetted with standards and samples. Any unbound substances in the wells were removed with wash buffer and an enzyme-linked polyclonal antibody specific for cytokine of interest was added to the wells thereafter. Washing steps were followed to remove unbound antibody-enzyme reagent. Finally, a substrate solution was added to the wells and the color development was stopped. The intensity of the color developed in proportion to the amount of bound cytokine of interest was measured in microplate reader (iMark; Bio-rad).

### Statistical analyses

For quantification, all samples were run in duplicate and three independent experiments were performed. Student’s t-tests or Mann-Whitney U tests were used to compare the differences between *Candida* only, bacteria only and *Candida* co-cultured with bacteria. A *P*-value of < 0.05 was considered statistically significant.

## Data Availability

We declare that all data is included in this manuscript and there are no supplimentary data files.

## Abbreviations

IL-8: Interleukin 8
TNF-α: Tumor necrosis factor alpha
ATCC: American Type Culture Collection
CCUG: Culture Collection University of Gothenburg
OD: Optical density
EDTA: Ethylenediamine tetra-acetic acid
HRP: Horseradish peroxidase
RT-PCR: Reverse transcription polymerase chain reaction
GAPDH: Glyceraldehyde 3-phosphate dehydrogenase
